# Pleomorphic Adenoma With Disrupted Biphasic Ductal Structures Attributed to Lymphoid Infiltration: A Case Report

**DOI:** 10.7759/cureus.72799

**Published:** 2024-10-31

**Authors:** Maiko Tsuchiya, Yoshinao Kikuchi, Toshitaka Nagao, Shiori Watabe, Yuya Shimizu, Yasutoshi Oshima, Yuko Sasajima, Hiroshi Uozaki

**Affiliations:** 1 Pathology, Teikyo University School of Medicine, Tokyo, JPN; 2 Anatomic Pathology, Tokyo Medical University Hospital, Tokyo, JPN; 3 Otolaryngology, Teikyo University School of Medicine, Tokyo, JPN

**Keywords:** hmga2, parotid gland, pleomorphic adenoma, saliary gland tumor, talp, tumor-associated lymphoid proliferation

## Abstract

Pleomorphic adenoma (PA) is a benign salivary gland tumor with diverse cytomorphological and architectural features, typically presenting biphasic ductal structures within a chondromyxoid matrix. We report a unique case of PA where dense lymphoid infiltration disrupted these structures, resulting in duct-like slit structures lined with a single layer of spindle cells, lacking the biphasic pattern. These spindle cells demonstrated myoepithelial nature, confirmed by positive immunostaining for pan-cytokeratin, S100 protein, and calponin. Fluorescence in situ hybridization analysis revealed HMGA2 rearrangement, supporting the diagnosis of PA. Unlike typical tumor-associated lymphoid proliferation (TALP), our case showed severe lymphocytic infiltration predominantly composed of CD8+ T cells, which eliminated mainly glandular epithelial cells. This case emphasizes highlights an unusual immune response in PA, broadening our understanding of its pathological spectrum.

## Introduction

Pleomorphic adenoma (PA) is the most common subtype among benign salivary gland tumors, accounting for 50-70% of all salivary gland tumors. PA is characterized by its cytomorphological and architectural diversity with biphasic tubular structures accompanied by a chondromyxoid or fibrous stromal component [1]. PA frequently harbors translocations or intrachromosomal rearrangements, resulting in gene fusions involving PLAG1 on 8q12 or HMGA2 on 12q14.3 (respectively in 50% and 10-15% of cases) [1,2]. Herein, we report a case of PA in which the predominantly luminal epithelial cells of biphasic ducts were observed to degenerate and disappear, presumably due to an immune response.

## Case presentation

**Figure 1:**
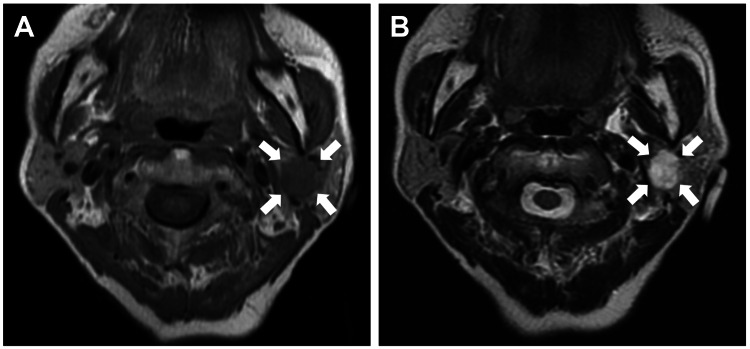
Magnetic resonance imaging (MRI) findings. MRI reveals a well-defined, oval-shaped tumor within left parotid gland on T1-weighted image (A) and T2-weighted image (B).

The patient was a 70-year-old woman who had noticed swelling in her left parotid gland for the past four years. Magnetic resonance imaging revealed a well-defined, oval-shaped tumor within the left parotid gland, exhibiting low-signal intensity on T1-weighted images and high-signal intensity on T2-weighted images (Figure 1). Tumor enucleation was performed.

**Figure 2:**
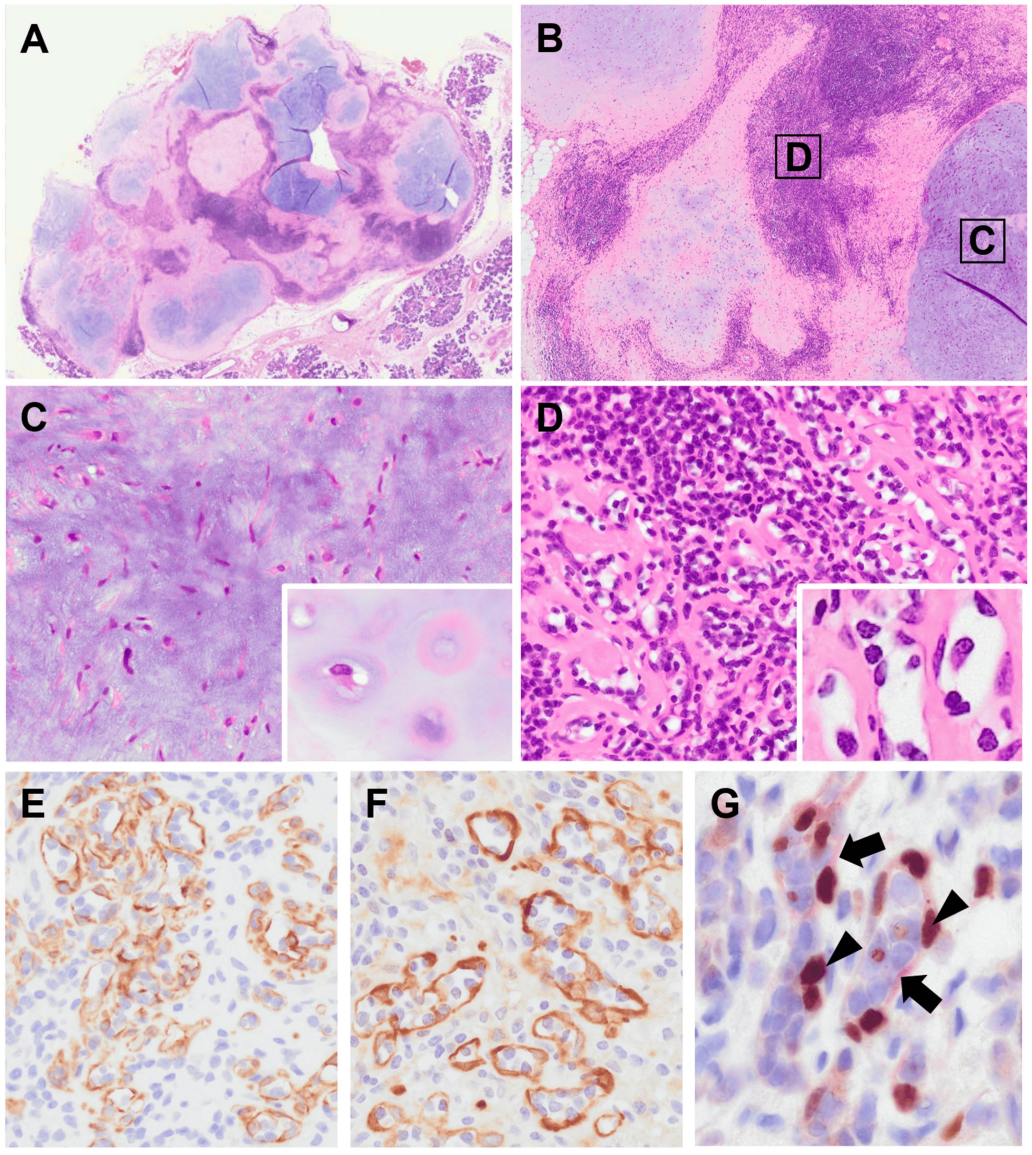
Histopathological findings of the tumor. (A) Low magnification view of the tumor reveals a well-demarcated mass. (B) Within the tumor, lobular chondromyxoid matrix components are observed (C), and dense lymphocytic infiltration is evident between the lobules (D). Labels 'C' and 'D' indicate the areas shown in high magnification in Figures 2C, 2D, respectively. (C) High-magnification image of chondromyxoid matrix area. Bland spindle to stellate tumor cells are sparsely distributed. Cartilage differentiation was focally evident (inset). (D) Slit duct-like structures are scattered in the area of severe lymphocytic infiltration. High-magnification image of duct-like slit structures lined by spindle cells (inset). The biphasic pattern is indistinct. Immunohistochemical staining for pan-cytokeratin (AE1/AE3) (E) and calponin (F). (G) Double immunostaining for S100 protein (red; arrow) and p63 (brown; arrowhead). These results indicate the myoepithelial cells lining the duct-like structures.

**Figure 3:**
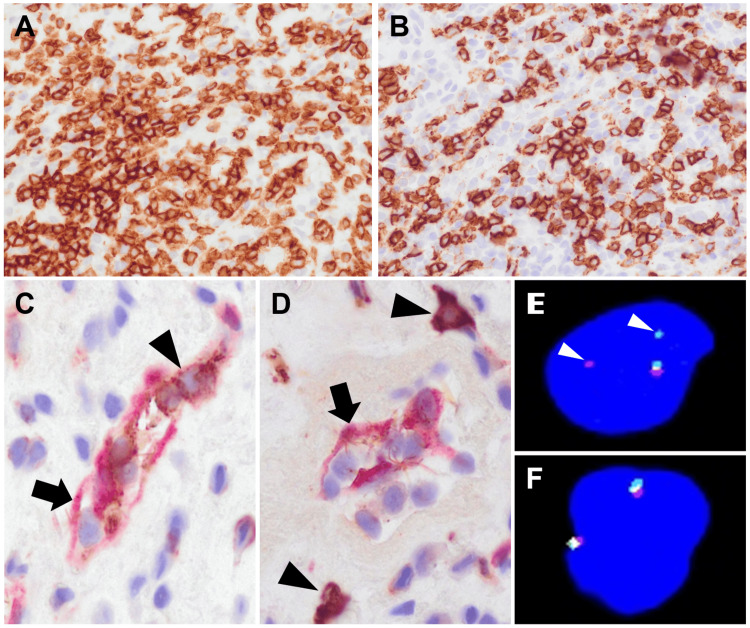
Immunohistochemistry focusing on lymphocytic infiltration and a break-apart fluorescence in situ hybridization (FISH) analysis. Immunohistochemical staining for CD3 (A) and CD20 (B). In the area of severe lymphocytic infiltration, CD3+ T cells are slightly predominant, although there was a mixture of both T cells and CD20+ B cells. (C) Double immunostaining for the S100 protein (red; arrow) and CD8 (brown; arrowhead). CD8+ T cells invade duct-like slit structures lined by S100-positive myoepithelial cells. (D) Double immunostaining for the S100 protein (red; arrow) and CD20 (brown; arrowhead). CD20+ B cells were found in areas unrelated to slit duct-like structures lined by S100-positive myoepithelial cells. The FISH analysis detected *HMGA2* rearrangement (E) but not *PLAG1* rearrangement (F).

Macroscopically, the tumor was a whitish mass with a maximum diameter of 13 mm. Histologically, the tumor was primarily composed of solid and lobular chondromyxoid matrix components (Figure 2A). A dense lymphocytic infiltrate was evident between the lobules of the chondromyxoid matrix with sparsely distributed bland spindle to stellate tumor cells (Figures 2B, 2C). Cartilaginous differentiation was also prominent in some areas (Figure 2C, inset). In areas of severe lymphocytic infiltration, a small number of duct-like slit structures were intermingled (Figures 2D, inset). The duct-like slit structures were lined with a single layer of spindle cells, which obscured the biphasic pattern. Immunohistochemically, most of the cells consisting of duct-like slit structures were positive for pan-cytokeratin (AE1/ AE3 and CAM5.2), S100 protein, calponin, and p63 indicating their myoepithelial nature (Figures 2E-2G). Some tumor cells were CK7 positive, but no apical staining for EMA, a luminal cell marker, was identified. Additionally, spindle or stellate cells in the myxoid area were positive for S100 protein. All tumor cells in both myxoid and lymphocytic infiltration areas were negative for PLAG1. Within areas of severe lymphocytic infiltration, both CD3+ T cells and CD20+ B cells infiltrated, but the former was predominant (Figures 3A, 3B). Both CD8+ T cells and CD20+ B cells infiltrated both the luminal and extraluminal areas of the duct-like slit structures. However, the majority of CD8+ T cells were localized within duct-like slit structures bordering S100-positive myoepithelial cells (Figure 3C). In contrast, few CD20+ B cells were observed within the luminal component (Figure 3D). A break-apart fluorescence in situ hybridization (FISH) analysis detected rearrangement of *HMGA2* but not *PLAG1* in myoepithelial cells (Figures 3E, 3F). Based on these histopathological findings and *HMGA2* rearrangement, the tumor was diagnosed as a PA. At 21 months after surgical excision, the patient remained free of recurrence.

## Discussion

Although tumor-associated lymphoid proliferation (TALP) is a lymphocytic reaction and a well-known finding in certain salivary gland tumors, its pathogenesis remains unclear. TALP is commonly found in mucoepidermoid carcinoma and acinic cell carcinoma; however, it is extremely rare in PA [3]. The degree and distribution of lymphocytic infiltration in TALP varies, ranging from mild and focal to severe and diffuse with lymphoid follicle formation accompanied by germinal centers [3]. In our experience, TALP may usually cause atrophy of tumor cell nests without the direct destructive findings observed in conditions such as MALT lymphoma or Sjögren's syndrome, where lymphoepithelial lesions are commonly present. This characteristic is also suggested by the existence of mucoepidermoid carcinoma cases with prominent TALP, such as the Warthin tumor-like variant, and by the diagnostic challenge of differentiating TALP from lymph node metastasis in certain salivary gland malignancies [4]. Since there is no strict definition of TALP, the lymphocytic infiltration observed in our case could also be interpreted as TALP; however, in that case, it may be an atypical form. In our case, it is hypothesized that infiltrating lymphocytes specifically attacked and eliminated the glandular epithelium within the biphasic ductal structures of PA, leaving only myoepithelial cells. Nevertheless, the possibility of just an atrophy of the biphasic duct cannot be excluded, as areas of hyalinization are observed.

Halo nevus (Sutton nevus) is characterized by an immune reaction against melanocytes, resulting in vitiligo around the nevus cell. A marked lymphocytic infiltrate causes partial or complete obscuration of the nevus cells [5]. Previous studies have shown that the lymphocytic infiltrate in halo nevus consists of approximately 80% T-lineage cells, primarily CD8+ T cells. Cytotoxic T cells, among CD8+ cells, release granzyme B or perforin, exhibiting cytotoxic activity [6,7]. These findings are highly comparable to those seen in our PA case. CD8+ T cells infiltrated the duct-like slit components, suggesting an immune reaction against glandular epithelial cells. It is considered that the immune response in this tumor differs from that seen in usual TALP and is more akin to the phenomenon observed in halo nevus.

As mentioned above, in malignant salivary gland tumors, TALP can pose a significant diagnostic challenge, especially when it mimics lymph node involvement, potentially leading to misdiagnosis [4]. Accurate differentiation between TALP and true lymph node involvement is crucial for proper tumor staging. Given that salivary glands often contain intraglandular lymph nodes, distinguishing between true lymph node involvement and TALP is necessary. Traditionally, features such as the lymph node capsule and subcapsular sinus have been used for this purpose, but these structures can sometimes be ambiguous. Recent studies suggest that immunostaining with low molecular weight cytokeratin (CAM5.2) is a reliable tool for this differentiation [4]. Lymph nodes contain extrafollicular reticulum cells (ERCs) that express low molecular weight cytokeratin, which are absent in TALP. Therefore, utilizing CAM5.2 immunostaining to identify ERCs can aid in accurately distinguishing TALP from true lymph node involvement, thereby reducing the risk of misdiagnosis. In our case, the duct-like slit structures were positive for CAM5.2, suggesting the possibility of myoepithelial differentiation. However, differentiation from lymph node metastasis is not diagnostically relevant for this case.

## Conclusions

Our case highlights that PA rarely demonstrates obscure glandular epithelial cells with marked lymphocytic infiltration, potentially because of an immune reaction mediated by CD8+ T cells against tumor cells. Recognizing this phenomenon is important for accurate diagnosis, especially when typical features of PA are obscured. Even in such cases, confirming the presence of rearrangements involving *PLAG1* or *HMGA2* could be helpful for making a diagnosis. Further documentation of similar cases may provide a deeper understanding of the interaction between PA and the immune system.
